# Long-term risk of shunt failure after brain tumor surgery

**DOI:** 10.1007/s10143-021-01648-5

**Published:** 2021-10-29

**Authors:** Sayied Abdol Mohieb Hosainey, Benjamin Lassen Lykkedrang, Torstein R. Meling

**Affiliations:** 1 Department of Neurosurgery, Bristol Royal Hospital for Children, Bristol, UK; 2 Department of Radiology, Hospital of Southern Norway, Kristiansand, Norway; 3 Faculty of Medicine, Institute of Clinical Medicine, University of Oslo, Oslo, Norway; 4 Department of Neurosurgery, Oslo University Hospital, Oslo, Norway; 5 Department of Neurosurgery, Geneva University Hospitals, Geneva, Switzerland; 6 Faculty of Medicine, University of Geneva, Geneva, Switzerland

**Keywords:** Brain tumor, Complications, Hydrocephalus, Shunt failure, VP shunt, Survival

## Abstract

Long-term risks and survival times of ventriculoperitoneal (VP) shunts implanted due to hydrocephalus (HC) after craniotomy for brain tumors are largely unknown. The aim of this study was to establish the overall VP shunt survival rates during a decade after shunt insertion and to determine risks of shunt failure after brain tumor surgery in the long-term period. In this population-based cohort from a well-defined geographical region, all adult patients (> 18 years) from 2004 to 2013 who underwent craniotomies for intracranial tumors leading to VP shunt dependency were included. Our brain tumor database was cross-linked to procedure codes for shunt surgery (codes AAF) to extract brain tumor patients who became VP shunt dependent after craniotomy. The VP shunt survival time, i.e. the shunt longevity, was calculated from the day of shunt insertion after brain tumor surgery until the day of its failure. A total of 4174 patients underwent craniotomies, of whom 85 became VP shunt dependent (2%) afterwards. Twenty-eight patients (33%) had one or more shunt failures during their long-term follow-up, yielding 1-, 5-, and 10-year shunt success rates of 77%, 71%, and 67%, respectively. Patient age, sex, tumor location, primary/repeat craniotomy, placement of external ventricular drainage (EVD), ventricular entry, post-craniotomy hemorrhage, post-shunting meningitis/infection, and multiple shunt revisions were not statistically significant risk factors for shunt failure. Median shunt longevity was 457.5 days and 21.5 days for those with and without pre-craniotomy HC, respectively (*p* < 0.01). This study can serve as benchmark for future studies.

## Introduction

Surgical resection of brain tumors is considered to be the primary choice of treatment for patients with debilitating neurological symptoms. The efficacy of craniotomy for brain tumors has been well established with regard to quality of life [2, 4, 28, 34] and prolongation of life [16, 20, 27, 28, 40, 43, 44]. Although the primary aim may be to cure disease or restore neurological function [7], risks of surgery such as infection [30], bleeding [15, 22, 30], surgical morbidity and mortality [2, 3, 30, 42], neurological deficits [5, 6, 25, 32], and changes to cerebrospinal fluid (CSF) dynamics leading to hydrocephalus (HC) and subsequent ventriculoperitoneal (VP) shunt dependency [18, 19] remain significant concerns to the neurosurgeon.

VP shunt insertion is the most commonly performed procedure for treatment of HC as it provides an immediate and effective diversion of accumulated CSF in the brain due to changes in CSF dynamics. Even though the main goal of shunting is to provide relief of intracranial pressure and improve symptoms, shunt failures remain a considerable challenge. Although studies of shunt failures with respect to the congenital conditions in the pediatric population [23, 26, 39], hemorrhage-related HC [12], idiopathic conditions [1, 35], and infections [26, 29] have been published, reports on risks of long-term shunt longevity after brain tumor surgery remain scarce in the literature [12, 37, 38].

In this large population-based study of all adult patients who underwent brain tumor surgery from a well-defined geographical region spanning a period of 10 years, we primarily wished to determine the 1-year, 5-year, and 10-year shunt failure rates in order to determine shunt longevities in the long-term period after craniotomy for brain tumors. The secondary endpoint was to identify possible risk factors of reduced long-term shunt longevity.

## Materials and methods

### Collection of data

In this population-based cohort, our prospectively collected database was reviewed to identify all adult patients operated at a single regional health care center between 2004 and 2013. The following patient demographics were recorded: age at time of shunt insertion, sex, status of hydrocephalus prior to craniotomy (yes/no), tumor location (supratentorial/infratentorial), intra-axial or extra-axial tumor location (established on imaging diagnostics reported by neuroradiologists), primary/repeat (secondary) tumor resection, histology, EVD placement (pre-craniotomy, simultaneously with craniotomy and post-craniotomy), ventricular opening during craniotomy (yes/no), post-craniotomy hemorrhage (yes/no), post-craniotomy meningitis/infection (yes/no), and number of shunt revision procedures with confirmed shunt failures. The first craniotomy in a specific location was defined as primary craniotomy and all subsequent craniotomies in the same location were defined as secondary. Therefore, a patient could have had more than one primary craniotomy, if operated on multiple/different locations. No patients were lost to follow-up.

In order to identify patients who underwent an EVD procedure before, during, and/or after craniotomy, and definitive VP shunting after brain tumor surgery, our tumor database was cross-linked with our surgical procedure code database using the Nordic Medico-Statistical Committee Classification of Surgical Procedures (NCSP) codes for CSF-related procedures (operation code AAF). Subsequently, ICD-10 codes (G91) were reviewed to verify each case. The criterion for EVD placement was presence of symptomatic HC requiring treatment to prevent further neurological deterioration and brain damage, either prior to craniotomy or concurrently with craniotomy. For those who developed HC post-craniotomy, an EVD was placed with an aim of avoiding permanent shunting. Those who had biopsies and those with pre-existing VP shunts prior to their craniotomies were excluded from this study.

Time from insertion of VP shunt to shunt failure was recorded. Suspicion of VP shunt failure was initially based on clinical signs and symptoms of altered intracranial pressure and radiological signs of ventricular enlargement as depicted on CT and/or MR imaging including T2/FLAIR-weighted sequences. A confirmed shunt failure was if/when patients underwent a shunt revision procedure resulting in a replacement of the whole shunt or in part by its individual components such as catheter replacement, as a result of blockage and/or change or replacement of shunt valve. Otherwise, if the shunt was only tested for functionality without any replacements, shunt malfunction was ruled out. All patients underwent either MRI or CT head imaging at time of suspected shunt failure.

For patients with confirmed shunt failure, we reviewed operation notes to determine whether the ventricles were opened during craniotomy for brain tumor in order to analyze this as a potential risk factor. We also recorded post-craniotomy hemorrhage (intraparenchymal and/or intraventricular) and infection (positive CSF and device cultures including CSF pleocytosis with clinical picture of infection requiring shunt removal) to analyze as risk factors for reduced shunt longevity.

The survival time of VP shunts, defined as VP shunt longevity, was calculated from the day of shunt insertion post-craniotomy for brain tumor until the day of confirmed shunt failure. VP shunt failure rates were determined at 1 year, 5 years, and 10 years after shunt insertion and risk factors were analyzed with regard to overall long-term shunt failure. Multiple VP shunt revisions were defined as shunt revision procedures ≥ 2 shunt revisions due to shunt failure. We excluded duplication of patient identification numbers (IDs) in order to avoid multiple counts of the same patient in our analyses and to account for multiple surgical interventions on the same patient. Hence, patient-to-craniotomy ratio was ensured to be 1:1 in the final analysis for long-term shunt longevity and risks of failure. As such, one patient could have multiple craniotomies and multiple shunt revision procedures for analyzing risks of reduced shunt longevity.

### Statistical analysis

For analysis of long-term VP shunt longevity, the Kaplan–Meier method was used to construct survival curves which were calculated from first day of VP shunt insertion to date of first revision or shunt removal. The Kaplan–Meier survival curves were also dichotomized with respect to patients with and without pre-craniotomy hydrocephalus in the 1-year and 5-year period. Log rank test was applied to determine statistical significance of different risk factors for shunt failure. Cox proportional hazard regression models were used to identify multiple potential predictor variables with respect to time to shunt failure. Chi-square (*χ*^2^) and Fisher’s exact test were used for comparison between categorical variables. Analysis of variance (ANOVA) and Student’s *t* test were used for continuous variables. Statistical significance was set at *p* < 0.05 and for all analyses the statistical software JMP (version 9.03) was used.

## Results

### Overall demographics

**Fig. 1:**
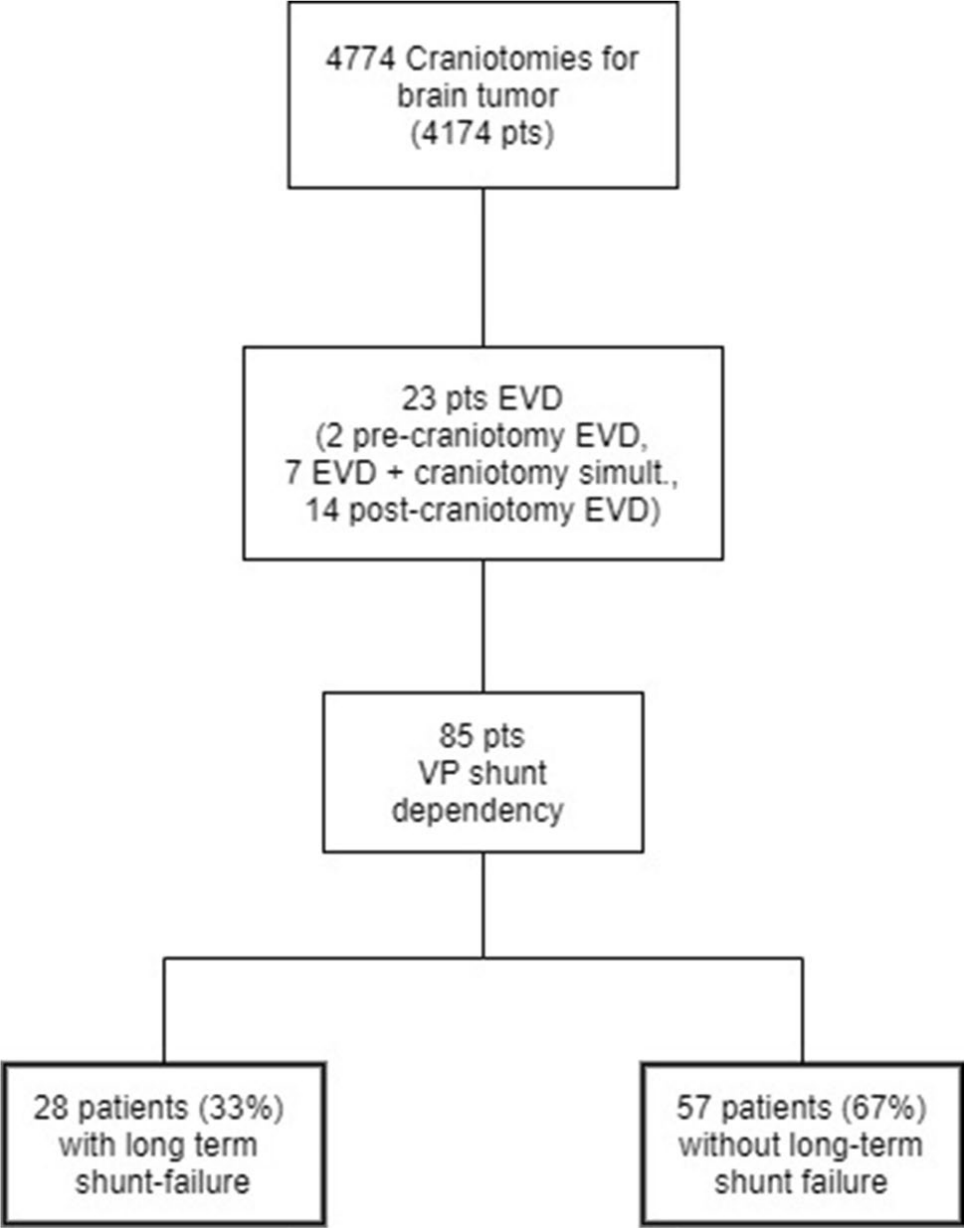
Flowchart illustrating all cases leading to VP shunt dependency and subsequently VP shunt failure within the study period

In total, 4774 craniotomies were performed on 4174 adult patients. There were 85 patients (2% of patients) who became VP shunt dependent after brain tumor surgery. Of these, 28 patients (33%) had shunt revision with confirmed shunt failure in the study period and constitute the study population (Fig. 1; Table 1). There were 13 males (46.4%) and 15 (53.6%) females with a median age at time of shunt failure of 61 years (range 26.1–79.8). Twelve patients (42.8%) had HC prior to craniotomy for brain tumor, while 16 patients (57.2%) did not. Tumors were located supratentorially in 25 patients (89.3%) and infratentorially in 3 patients (10.7%). Twenty-one patients (75%) underwent primary tumor surgery and 7 patients (25%) had repeat craniotomy for brain tumor. From the 28 patients with confirmed shunt failure, 3 patients (10.7%) had EVD concomitantly with tumor resection and 3 patients (10.7%) had post-craniotomy EVD. Nine patients (10.6%) had ventricular opening during tumor resection, one (3.6%) of whom had a short shunt longevity. None of those with shunt failure had post-craniotomy hemorrhage and only 2 patients (7.1%) had shunt infections. Nine patients (32.1%) had more than one shunt revision procedure (Table 1).

**Table 1:** Overview characteristics of patients with post-craniotomy VP shunt dependency and reduced shunt longevity

	**Total VP shunt dependency after craniotomy (*N*/%)**	**VP shunt failure (*N*/%)**	**No VP shunt failure (*N*/%)**
Total	85	28	57
Age (median years)	61.9	61.0	62.7
Sex			
Male	44 (51.8)	13 (46.4)	27 (47.4)
Female	41 (48.2)	15 (53.6)	30 (52.6)
Pre-craniotomy hydrocephalus			
No	46 (54.1)	16 (57.2)	30 (52.6)
Yes	39 (45.9)	12 (42.8)	27 (47.4)
Tumor location			
Supratentorial	68 (80.0)	25 (89.3)	47 (82.5)
Infratentorial	17 (20.0)	3 (10.7)	10 (17.5)
Extra-axial tumor	33 (38.8)	14 (50.0)	19 (33.3)
Intra-axial tumor	52 (61.2)	14 (50.0)	38 (66.7)
Surgery			
Primary	64 (75.3)	21 (75.0)	43 (75.4)
Secondary	21 (24.7)	7 (25.0)	14 (24.6)
Histology			
HGG	21 (24.7)	6 (21.5)	15 (26.3)
Meningioma	21 (24.7)	9 (32.2)	12 (21.1)
Metastasis	18 (21.2)	4 (14.3)	14 (24.6)
Other tumors	8 (9.5)	2 (7.1)	6 (10.5)
Ependymoma	4 (4.7)	2 (7.1)	2 (3.5)
Craniopharyngioma	4 (4.7)	1 (3.6)	3 (5.3)
Schwannoma	3 (3.6)	2 (7.1)	1 (1.7)
Choroid plexus tumor	2 (2.3)	0	2 (3.5)
Pituitary adenoma	2 (2.3)	0	2 (3.5)
LGG	2 (2.3)	2 (7.1)	0
EVD			
Pre-craniotomy EVD	2 (2.3)	0	2 (3.5)
EVD + craniotomy simultaneously	7 (8.2)	3 (10.7)	4 (7.0)
Post-craniotomy EVD	14 (16.5)	3 (10.7)	11 (19.2)
Ventricular entry during craniotomy	9 (10.6)	1 (3.6)	8 (14.0)
Post-craniotomy bleeding	8 (9.4)	0	8 (14.0)
Post-craniotomy infection	4 (4.7)	2 (7.1)	2 (3.5)
Multiple (≥ 2) shunt revisions	–	9 (32.1)	0

*EVD*, external ventricular drainage; *HGG*, high-grade glioma; *LGG*, low-grade glioma; *VP*, ventriculoperitoneal

### Long-term shunt longevity

**Fig. 2:**
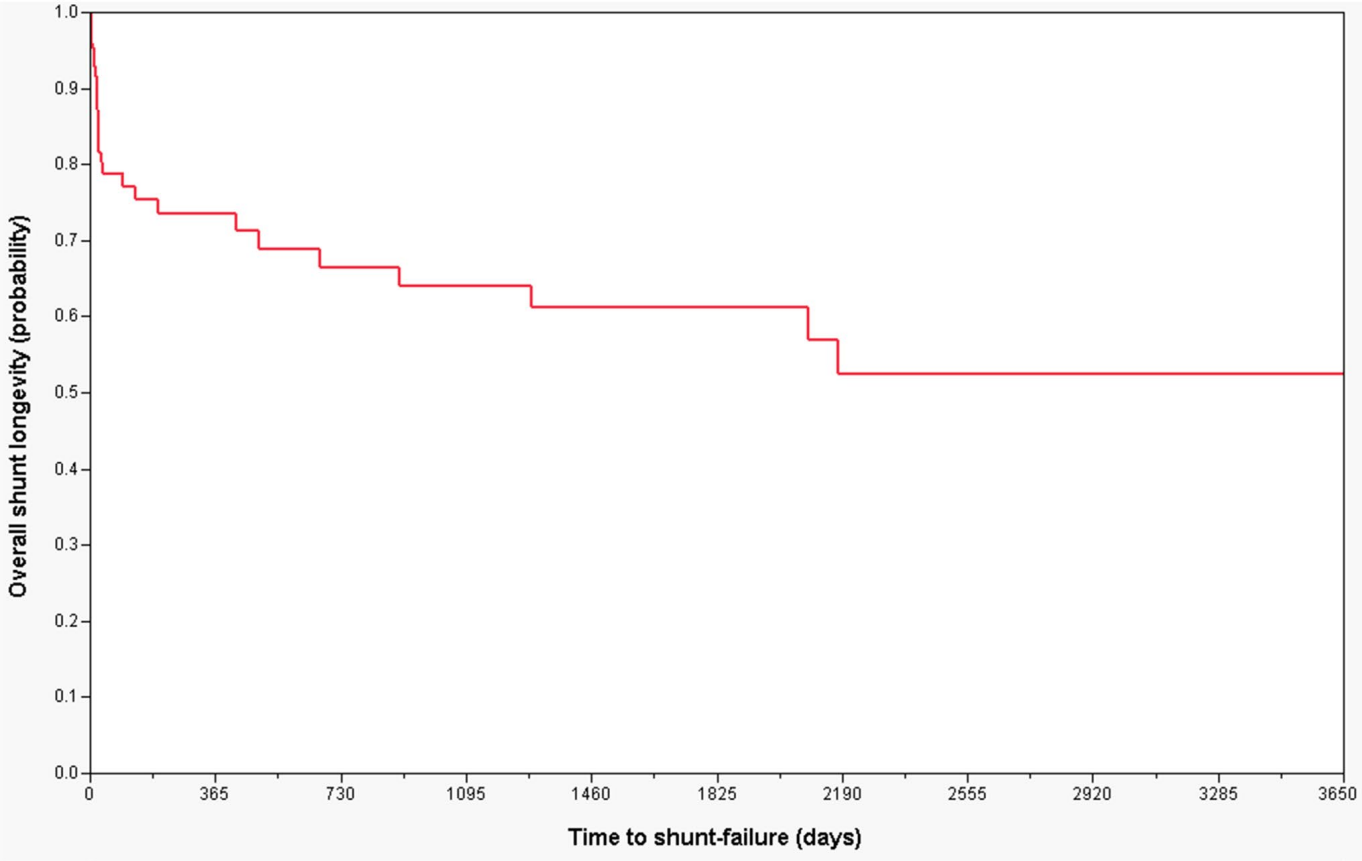
Kaplan–Meier curve showing overall 10-year shunt longevity for all patients in the entire study period

Overall, there were 65, 60, and 57 out of 85 patients who did not have any shunt malfunction at 1 year, 5 years, and 10 years, respectively, yielding VP shunt success rates of 77%, 71%, and 67% (Fig. 2; Table 2). The median shunt longevity was 20.5, 23, and 23.5 days, at 1 year, 5 years, and 10 years, respectively (Fig. 2; Table 2).

**Table 2:** Shunt longevity time frames of selected variables after craniotomy for brain tumor

	**1 year post VP shunting**	**5 years post VP shunting**	**10 years post VP shunting**
Patients with shunt failure (*N*)	20	5	3
Shunt failure rate (cumulative %)	23	29	33
Sex (*N*/%)			
Male	12 (60)	1 (20)	0
Female	8 (40)	4 (80)	3 (100)
Shunt longevity days^a^	20.5	23.0	23.5
HC prior to craniotomy (*N*/%)			
Yes^b^	5 (25)	4 (80)	3 (100)
No^c^	15 (75)	1 (20)	0
Tumor location (*N*/%)			
Supratentorial	18 (90)	4 (80)	3 (100)
Infratentorial	2 (10)	1 (20)	0
Intra-axial (*N*/%)	9 (45)	3 (60)	2 (67)
Extra-axial (*N*/%)	11 (55)	2 (40)	1 (33)
Surgery (*N*/%)			
Primary	15 (75)	3 (60)	3 (100)
Secondary	5 (25)	2 (40)	0

^a^Time given as median unless otherwise specified

^b^Cases with persisting postoperative HC (after craniotomy) requiring VP shunting

^c^Cases with de novo (new onset) postoperative HC requiring VP shunting

*HC*, hydrocephalus; *VP*, ventriculoperitoneal

**Fig. 3:**
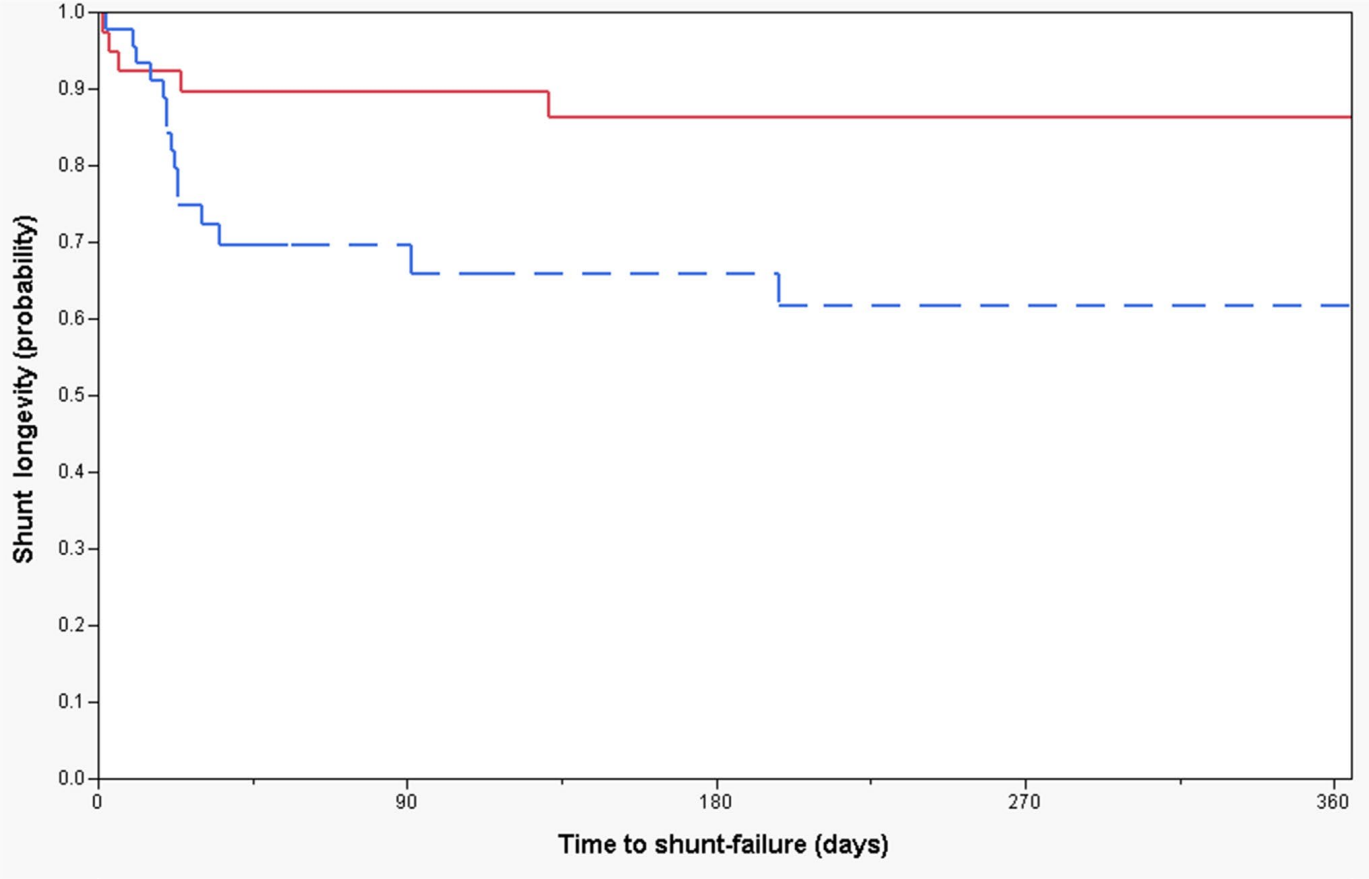
Kaplan–Meier curves demonstrating 1-year shunt longevity. Red continuous and blue dotted lines represent patients with and without pre-craniotomy hydrocephalus, respectively

**Fig. 4:**
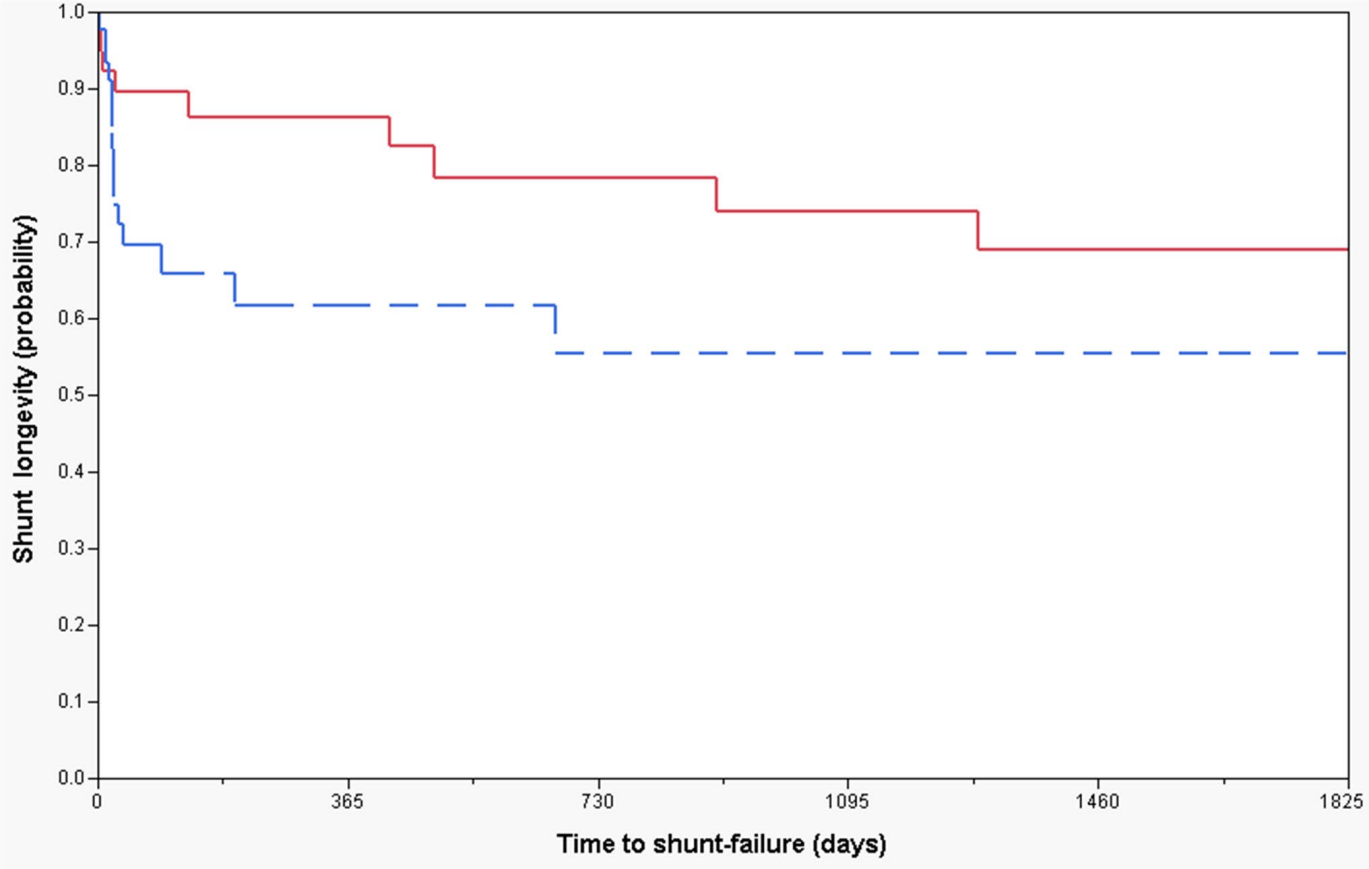
Kaplan–Meier curves demonstrating 5-year shunt longevity. Red continuous and blue dotted lines represent patients with and without pre-craniotomy hydrocephalus, respectively

Median shunt longevity was 457.5 days and 21.5 days, respectively, for those with and without pre-craniotomy HC (Figs. 3 and 4; Table 3). Patients with pre-craniotomy HC had significantly lower risk of shunt failure overall in the long term in both univariate (HR 0.3, CI [0.1–0.7], *p* < 0.01) and multivariate analysis (HR 0.1, CI [0.1–0.5], *p* < 0.05) compared to patients without pre-craniotomy hydrocephalus (Figs. 3 and 4; Table 3).

**Table 3:** Long-term shunt longevity and risk analysis of shunting with univariate and multivariate proportional hazards ratio model

	**Risk of long-term shunt failure**	
	Univariate (HR, CI [95%])	Multivariate (HR, CI [95%])
Age at time of shunt failure	1.0 [0.9–1.1]	1.0 [0.9–1.1]
Sex		
Male	1	1
Female	0.5 [0.2–1.2]	0.4 [0.1–1.2]
Pre-craniotomy HC		
No	1	1
Yes	0.3 [0.1–0.7] ^a^	0.1 [0.1–0.5] ^b^
Tumor location		
Supratentorial	1	1
Infratentorial	0.8 [0.2–2.3]	0.4 [0.1–2.5]
Intra-axial tumor	1	1
Extra-axial tumor	0.9 [0.4–2.0]	0.8 [0.3–2.5]
Surgery		
Primary	1	1
Secondary	1.1 [0.4–2.3]	0.4 [0.1–1.7]
EVD treatment		
Pre-craniotomy EVD	–^c^	–^c^
EVD + craniotomy simultaneously	1.1 [0.3–3.2]	4.8 [0.6–41.7]
Post-craniotomy EVD	0.8 [0.2–2.4]	0.8 [0.2–2.9]
Ventricular opening at craniotomy		
No	1	1
Yes	1.2 [0.1–6.1]	1.2 [0.1–10.4]
Post-craniotomy hemorrhage		
No	–^c^	–^c^
Yes	–^c^	–^c^
Post-craniotomy meningitis/infection		
No	1	1
Yes	8.5 [0.5–66.3]	7.6 [0.8–49.7]
Multiple revisions (≥ 2 procedures)		
No	1	1
Yes	1.3 [0.5–2.8]	0.4 [0.1–2.5]

^a^*p* < .01

^b^*p* < .05

^c^Too few cases in variable parameters to determine HR for long-term shunt longevity

### Risk factors for reduced long-term shunt longevity

Neither age nor sex was significantly associated with long-term shunt failure in univariate or multivariate analysis (Table 3). Similarly, tumor location (dichotomized into supratentorial/infratentorial compartment and intra-axial/extra-axial tumors), primary/repeat craniotomy, EVD placement (prior to, simultaneously with, and after craniotomy), ventricular entry, post-craniotomy hemorrhage, post-shunting meningitis/infection, and multiple shunt revisions/failures were not significantly associated with reduced shunt longevity in the long-term period (Table 3).

## Discussion

The innate natural history of brain tumors or their surgical interventions may lead to HC that necessitates temporary CSF diversion procedures, such as external ventricular drainage (EVD) and/or endoscopic third ventriculostomy (ETV), or lead to permanent CSF diversion. Although the incidence and risk factors for development of postoperative HC leading to VP shunt dependency of patients with and without HC prior to a craniotomy for brain tumor have been previously described [18, 19], the long-term outcomes of VP shunts in brain tumor patients are largely unknown.

In our study, a total of 85 patients in a consecutive cohort of 4174 adult patients became VP shunt dependent after craniotomies for brain tumors (2% of patients). Of these, 28 patients (33%) had confirmed shunt failures during the study period of 10 years, yielding cumulative shunt success rates at 1, 5, and 10 years of 77%, 71%, and 67%, respectively (Fig. 2; Table 2). In the literature, reports of long-term shunt failure rates in the adult population range from 11% at 1 year up to 34% at 10 years [24, 29, 37, 46], with the majority of these consisting of various underlying etiologies including congenital diseases, normal pressure hydrocephalus, trauma, tumor, and intracranial cysts. In a nationwide study on adult HC patients, Donoho et al. [12] found that 9% of patients required a shunt revision with a median time to shunt revision of only 41 days. However, their study included shunts due to all underlying conditions and 45% had shunt insertions due to obstructive HC. Furthermore, the authors did not state whether an obstructive HC was due to brain tumors and their shunt revision rates reflect the first 6 months only. In another study by Reddy et al. [37] on VP shunt complications in HC patients with intracranial tumors, 20% and 24% of patients experienced shunt failures requiring shunt revisions within 1 year and 5 years, respectively. Our lower shunt failure rates might be explained by inclusion of brain tumor patients in adults only. However, the overall median time to shunt failure was shorter in our study as compared to that of Donoho et al. [9], but this might be explained by tumor debris and higher protein content in the CSF of patients with brain tumors leading to shunt blockage compared to other non-oncological conditions.

Recently, Hosainey et al. [17] studied risk factors of early VP shunt failure after brain tumor surgery and found that patients with pre-existing, non-treated HC prior to craniotomy had a significantly shorter shunt-free period before definitive shunting compared to those without pre-craniotomy HC. Interestingly, in the current study, shunted patients who had HC prior to craniotomy had significantly longer shunt survival (Figs. 3 and 4). This indicates that in patients with distinct pathologies and profoundly deranged CSF dynamics in the early postoperative course after brain tumor surgery, early VP shunting may serve as “prophylaxis” against further CSF disturbances in the future and hence give prolonged shunt longevity due to early “normalized” hydrodynamics by shunting. The median shunt longevity was 457.5 days and 21.5 days for those with and without untreated HC pre-craniotomy, respectively (Figs. 3 and 4). In the literature, median shunt survival times range from 19 days in the short term up to 20.1 years in the long term [8, 12, 24, 29, 36]. However, these studies were not limited to brain tumor patients and include a plethora of underlying conditions. Early changes to CSF dynamics as a result of overloading venous outflow and CSF pathway obstruction caused by disease burden have been described in the literature [41, 45]. Further neuronal cell death may also ensue [10, 11] if the disease process is left untreated. This requires early VP shunt insertion in order to normalize intracranial processes and avoid brain damage caused by a disrupted hydrocephalic state. Nonetheless, another plausible explanation may be that although some patients with pre- and post-craniotomy HC underwent shunting in the early postoperative course, they might have experienced spontaneous resolution of their hydrocephalic condition in the long-term period. Therefore, a “silent” shunt obstruction may have ensued, making them effectively shunt independent, wherefore a shunt obstruction would be unnoticed due to lack of signs and symptoms.

Neither patient age at time of shunt placement nor sex was associated with reduced shunt longevity (Table 3). In a study by Reddy et al. [37] of VP shunt complications for hydrocephalus in patients with intracranial tumors, males had significantly lower 3- or 6-month survival rates compared to females (*p* < 0.001). This is in contrast to our findings. They also reported a 2% decrease in odds of shunt failure with increasing age at time of shunt insertion [37]. Comparatively, some studies have associated younger age with higher risk of shunt failure [12, 37], whereas others have not reached this conclusion regarding age in the short-term [1, 13, 17] nor in the long-term period after shunting [24].

Tumor location was not significantly associated with reduced shunt longevity despite dichotomizations into supratentorial/infratentorial and intra-axial/extra-axial tumor location (Table 3). Although somewhat surprising, this is in line with previous studies that did not find extra-axial/intra-axial tumor location to be significantly associated with early shunt failure after craniotomy for brain tumor [17]. In contrast, Khan et al. [24] studied factors affecting shunt survival in adults and found that extra-axial tumors were more common (13.2%) than intra-axial tumors (9.7%), but in line with our results, they reported that brain tumor location was not a significant risk factor of shunt failure.

With respect to tumor histology, several extra-axial tumors such as choroid plexus tumors, craniopharyngiomas [19] and schwannomas [14], and periventricular intra-axial tumors [21] have been reported to have increased risk of postoperative HC and shunt dependency. Additional stratified risk analysis into those with and without pre-craniotomy HC did not reveal intra-axial/extra-axial tumors as statistically significant risk factors for reduced long-term shunt longevity in our study. Nonetheless, similar reports are scarce in the literature making comparative analysis to our study difficult.

In our study, meningiomas had the highest incidence of shunt failure during follow-up (Table 1). Interestingly, these are extra-axial tumors and not usually located in the ventricles, but they might cause significant CSF dynamics changes after craniotomy if the tumor volume is large, particularly in the posterior fossa region, or if the area of resected/coagulated dura is large. Reddy et al. [37] reported that patients with benign tumors had higher risk of shunt revision, probably because of a shorter survival rate among patients with malignant brain tumors. In the abovementioned study by Khan et al. [24], the effect of brain tumor histology did not reach statistical significance (*p* = 0.062). In the same vein, Rinaldo et al. [38] found no difference in the incidence of shunt revision surgery in high-grade glioma patients as compared to NPH patients. We believe that the lower number of malignant brain tumor patients with reduced shunt longevity in our study might be due to the short overall survival of these patients, rendering shunt procedures futile when they present at advanced stages in the disease process. In addition to clinical diagnosis of shunt dysfunction, these patients may also suffer from ventriculomegaly as a consequence of radiation-induced brain atrophy, which is diagnosed radiologically. Lastly, patients with high-grade gliomas invariably see clinical deterioration due to tumor progression and a shunt dysfunction in this context may be overlooked.

In our study, primary/secondary surgery for brain tumor was not significantly associated with increased risk of reduced long-term shunt longevity (Table 3). Secondary/repeat surgery has been reported as a risk factor for postoperative HC and subsequent VP shunt dependency in patients with pre-craniotomy hydrocephalus [19] and one would expect repeat surgical intervention for recurrent brain tumor to cause even more CSF disturbance and shunt failures. However, only seven patients in our study cohort underwent repeat craniotomy for brain tumor, leaving a low statistical power and a high risk of a statistical error type II.

Placement of EVD for treating HC, regardless of timing before, during, or after craniotomy for brain tumor, was not associated with shunt longevity in the long term (Table 3). For shorter time periods, other studies have also reported no significant association between EVD placement and shunt failure within 30 days [31] and 90 days [17]. However, previous EVD placements and males have been reported to be risk factors for first revision for mechanical dysfunction, although the cause of HC had no impact on risk of shunt dysfunction [29]. Nevertheless, some of these studies have not been limited to brain tumors only, whereas our study includes all craniotomies for brain tumor irrespective of tumor histology.

Only 2 patients (7.1%) had shunt infection during the follow-up (Table 1). Although post-shunting meningitis/infection was not significantly associated with reduced shunt longevity (Table 3), infection has been shown to be associated with higher risk of shunt failure in some studies [29]. Our rates of infection lie in the upper range of published reports [1, 24, 26, 29], which can be explained by our inclusion criteria of adult patients with brain tumors only. However, the number of patients with infection was too few for adequate statistical power, possibly giving rise to false negative results in our study. Most of the shunt revisions happened during the first year after shunt insertion (Table 2). Whereas some have reported shunt failures in the first 6 months [13, 26, 29, 36], others have reported within the first year [33].

Nine patients in the study cohort (32.1%) underwent multiple shunt revisions (≥ 2 revisions) (Table 1), in keeping with other reports [46]. Korinek et al. [29] reported that previous shunt revision was an independent risk factor for infection leading to failure and Reddy et al. [37] reported single shunt revision procedures in 25 patients (13.4%) and multiple shunt revisions in 27 patients (14.4%) after initial shunt placement. Reddy et al. [37] also found that odds for multiple revisions among those with shunt system replacements were significantly higher (OR 24.39, *p* < 0.01) than those without any shunt replacement. They also showed that infection, shunt valve replacement, and externalization were also significantly associated with multiple revisions. However, the significance was lost when the data was adjusted for the effects of other risk factors such as shunt system replacement and proximal shunt complication. Our study did not find that multiple revision procedures (≥ 2 revision surgeries) were significantly associated with reduced shunt longevity in the long term (Table 3).

### Strength and limitations of the study

Our centralized neurosurgical health care center at Oslo University Hospital (Rikshospitalet and Ullevål) has a population-based referral of patients from a well-defined geographical region of Norway with approximately 2.8 million inhabitants. This reduces possible confounding effects of differences in access to health care services. As there is only one main neurosurgical department performing neurosurgical procedures from a defined geographical area, selection bias is avoided, which is inherently present in multicenter studies. Our study is unique in that we did not find any other large-scale studies with focus on analysis of long-term shunt longevity and possible risks associated with shunt failure after craniotomies for brain tumors where all patients are included regardless of tumor histology. There is no selection bias, as the study includes all craniotomies performed within the study period from a histologically verifiable intracranial tumor. The design of our study is a retrospective analysis of a prospectively collected database. Additionally, by cross-linking our tumor database with the registry for neurosurgical procedures, we have included all craniotomies leading to shunt dependency and have been able to perform shunt survival and risk analysis as per our main aims of the study. Finally, no patients have been lost to follow-up and to the extent of our knowledge, this is the largest study with respect to analyzing long-term shunt survival and risks associated with shunt failure in patients whom became shunt dependent after craniotomy for brain tumors.

The foremost limitation of this study is its retrospective analysis of prospectively collected data. Surgeon’s preferences with regard to treating hydrocephalus and timing of shunt revision might be a potential selection bias. Other variables such as tumor volume, shunt valve type, and whether proximal or distal catheter malfunction/block was the cause of shunt failure were not included in our analyses. The analysis of images with regard to shunt failure was not performed in an automatized manner, due to lack of comparability across the different imaging modalities in absence of age-adjusted normal values and due to lack of comparability across the different imaging modalities. Even though CT/MRI was available for all patients included in the study, the presence or absence of ventriculomegaly leading to shunt failure and subsequent revision may have been limited by human error. Adjuvant treatments such as radiotherapy and chemotherapy and coexisting comorbidities were not included in the analyses, which may contribute to the risks and rates associated with reduced shunt longevity in the long-term period. Although being a large study, the number of patients might be so low in the final analyses giving rise to statistical type I and II errors, thus failing to identify true prognostic factors for shunt failures. As most published reports in the literature are biased with limitations to certain patient groups and tumor histologies and accounting for overall shunt failure rates, comparative analysis to our study was difficult. Our study was confined only to adults who had undergone craniotomies for brain tumors.

## Conclusion

The overall 10-year shunt success rate after brain tumor surgery was 67%. Median shunt longevities were 457.5 days and 21.5 days in those with and without pre-craniotomy hydrocephalus. Patients with pre-craniotomy hydrocephalus had significantly longer shunt longevity than those without pre-craniotomy hydrocephalus. Early “prophylactic” shunting of patient with persisting hydrocephalus after brain tumor surgery may yield prolonged shunt longevity in the long term. This study can serve as benchmark for future studies.

## Data Availability

Data may be given upon reasonable request.
